# The Effect of the Equivalent Permittivity Model in Contactless MIMO-GPR Imaging

**DOI:** 10.3390/s26051463

**Published:** 2026-02-26

**Authors:** Gianluca Gennarelli, Ilaria Catapano, Francesco Soldovieri

**Affiliations:** Institute for Electromagnetic Sensing of the Environment, National Research Council of Italy, Via Diocleziano 328, 80124 Napoli, Italy; catapano.i@irea.cnr.it (I.C.); soldovieri.f@irea.cnr.it (F.S.)

**Keywords:** ground-penetrating radar, linear inverse scattering, multiple input–multiple output

## Abstract

Multiple-Input–Multiple-Output Ground-Penetrating Radar (MIMO-GPR), collecting multiview–multistatic data, is now becoming an assessed diagnostic tool, enabling enhanced reconstruction accuracy and subsurface target detection due to the exploitation of multiple Tx/Rx channels. In this context, the present work deals with a 2D radar imaging approach for contactless MIMO GPR based on the equivalent permittivity concept. The imaging problem is formulated as a linearized inverse scattering problem under Born approximation, and a ray propagation model, based on equivalent permittivity spatially varying along depth, is adopted to account for the wave propagation through the air–soil interface. The resulting linear inverse problem is solved by means of an adjoint inversion, enabling reliable target reconstruction. Despite the approximation introduced by the present formulation, numerical simulations show that the proposed imaging strategy is sufficiently accurate from an engineering viewpoint and is computationally efficient.

## 1. Introduction

Ground Penetrating Radar (GPR) operating in a Multiple-Input–Multiple-Output (MIMO) configuration [1,2,3,4,5,6,7,8] is an advanced technology for non-destructive subsurface sensing, with applications in several fields such as infrastructure monitoring, archaeological prospection, utility detection, and many others. MIMO GPR systems are capable of simultaneously acquiring data from multiple transmitting/receiving (Tx/Rx) antennas. Such a configuration offers notable advantages with respect to standard multi-monostatic or Single-Input–Single-Output (SISO) systems made of one Tx/Rx antenna pair. Indeed, the MIMO configuration enables a significant illumination/observation diversity with lower survey times and improved target detection capabilities [8]. The use of multiple antennas enables the collection of multiview–multistatic data, providing a rich dataset that captures the subsurface target’s response from various viewing angles, thus enhancing the information content available for the imaging task.

However, novel challenges arise due to the need for advanced data processing algorithms capable of fully exploiting the multi-channel information. In relation to this aspect, the interpretation of MIMO GPR measurements necessitates ad-hoc inversion techniques capable of transforming the raw data into reliable subsurface images.

Through the years, different migration techniques have been proposed to focus MIMO GPR data, such as diffraction stacking [9], Kirchhoff migration [10], range migration [11], and reverse time migration [12]. An interesting comparison between Kirchhoff migration, phase shift migration, and frequency–wavenumber (f-k) or Stolt’s migration was reported in [13], showing that Kirchhoff migration has the best capability in resolving the targets at the expense of a higher sidelobe level and lower computational speed. In this respect, the most efficient algorithm is f-k migration, which can only handle the homogeneous medium. A modified backprojection (delay and sum) approach based on the coherent combination of the reconstructions achieved for a single-input multiple-output array was proposed in [14] in relation to the detection of landmines from forward-looking GPR data. Compressive sensing and sparse reconstruction strategies have also been developed due to their ability to exploit the inherent sparsity of many subsurface targets, enabling enhanced resolution from less data [15,16].

Another class of reconstruction approaches is provided by microwave tomography (MWT), which formulates the subsurface imaging task as an electromagnetic inverse scattering problem and provides a flexible tool to easily incorporate detailed forward models of wave propagation in inhomogeneous media in the imaging problem formulation.

Referring the reader to [17,18], and references therein for a detailed discussion, we recall that MWT approaches based on exact scattering models involve solving a non-linear and ill-posed problem. These approaches, also known as full-waveform inversion, are capable of retrieving both the geometrical and electromagnetic features of the targets (quantitative reconstruction). However, non-linear inversion still remains challenging in realistic GPR applications owing to known reliability issues (e.g., false solutions) [19], high computational costs associated with iterative optimization procedures, stringent requirements for accurate initial model estimates, and sensitivity to noise and model errors. Furthermore, the forward electromagnetic model requires an exact knowledge of the probing field, accounting for the interaction of the Tx antenna with the medium, which is often difficult to achieve in real cases and raises a problem of complexity comparable to the inverse scattering problem [20].

Conversely, imaging approaches based on approximate electromagnetic scattering models, e.g., those based on the Born approximation (BA), are classified as qualitative reconstruction methods. BA-based approaches can provide a suitable estimate of geometrical and electromagnetic features of the unknown targets (quantitative reconstruction) only when the latter satisfy the conditions under which the approximation is valid (see [17,18]). However, in general, they provide qualitative information (target position and approximate shape), and Linear MWT (LMWT)-based methods have been widely and successfully used in on-field GPR surveys and offer considerable versatility in processing data acquired from diverse measurement geometries and antenna array configurations [3,7,8,21]. Furthermore, BA-based approaches allow reconstruction performance to be evaluated as a function of the scenario (background medium, measurement configuration) and the effect of uncertainties in the model parameters to be assessed (e.g., errors in background permittivity or sensor positioning). The prediction of the expected imaging capabilities represents a key element required to set up the measurement configuration properly and facilitate the correct interpretation of the GPR imaging results.

In this regard, a 2D LMWT approach for MIMO-GPR working in down-looking contactless mode was recently developed by the authors in [3]. The approach exploits a ray-based propagation model describing the electromagnetic wave refraction at the air–soil interface and provides accurate subsurface target reconstructions. However, a shortcoming of the approach is related to the computational load of the discretization of the scattering operator, which rapidly grows with the size (in terms of wavelength) of the measurement/investigation domain. To overcome this limitation, this work proposes a 2D LMWT imaging approach based on the equivalent permittivity (EP) concept [22]. Such a concept was originally developed for SISO GPR in underwater imaging applications and subsequently generalized to inhomogeneous media characterized by a vertical permittivity gradient [23].

This paper presents an imaging approach based on an approximate scattering model (i.e., the EP model) that significantly simplifies the processing of contactless MIMO GPR data. The imaging problem is efficiently solved through the use of the linear scattering operator’s adjoint. The main objective is to analyze the system’s point spread function (PSF) to assess the impact of the EP model on imaging results compared to the ray-based model in [3]. Furthermore, this paper provides results of numerical experiments based on synthetic full-wave data.

The remainder of the paper is organized as follows. Section 2 describes the ray-based LMWT approach and provides the formulation of the equivalent permittivity model. Section 3 deals with the analysis of the imaging performance achievable through the equivalent permittivity model and reconstruction results based on full-wave data. Concluding remarks follow in Section 4.

## 2. LMWT Approach

**Figure 1:**
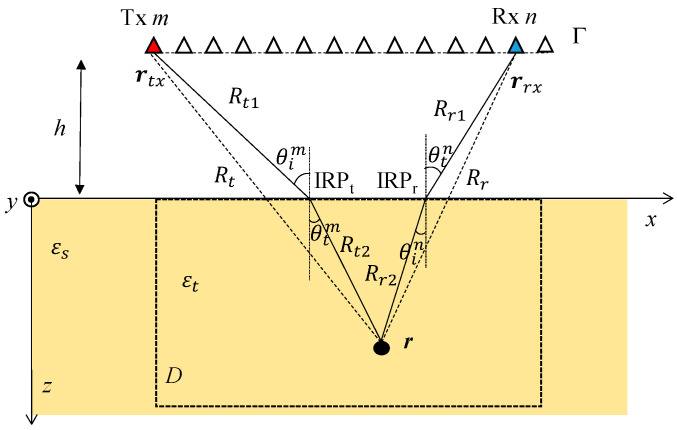
Geometry of the GPR imaging problem. The ray emitted by the *m*-th Tx antenna undergoes refraction at IRP_t_, impinges on the target, and is retransmitted in the air at IRP_r_, finally reaching the *n*-th Rx point. $\theta_{i}^{m}$ and $\theta_{t}^{m}$ are the incidence and transmission angles along the forward ray path. $\theta_{i}^{n}$ and $\theta_{t}^{n}$ are the incidence and transmission angles along the return ray path.

This section describes the scattering model underlying the LMWT approach. To this end, we consider the 2D reference scenario depicted in Figure 1, featuring a two-layered medium where the upper half-space (z < 0) is air and the lower half-space (z > 0) is the soil. The lower half-space is assumed to be a homogeneous, lossless, and non-magnetic medium with dielectric permittivity $\varepsilon_{s}=\varepsilon_{0}\varepsilon_{rs}$ ($\varepsilon_{0}$ = 8.85 × 10^−12^ F/m is the free-space permittivity and $\varepsilon_{rs}$ is the soil’s relative permittivity). The targets are supposed to reside in the investigation domain *D* and are described by the unknown dielectric permittivity function ε(r), with r being a generic point in *D*. The scene is probed by a MIMO GPR consisting of *M* transmitting (Tx) and *N* receiving (Rx) antennas arranged into a linear array covering the domain Γ and placed at a distance *h* above the air–soil interface (*z* = 0). The radar sensing process involves the sequential activation of the *m*-th Tx antenna, m=1, …, M, which illuminates the scene, and the scattered field is simultaneously collected at *N* measurement points. The antennas are modeled as filamentary *y*-directed electric currents of infinite extent and invariant along the *y*-axis (TM polarization), and operate in the angular frequency range Ω= [$\omega_{min},\omega_{max}$]. For simplicity, the pattern of the Rx antenna is neglected in the formulation; this means that the scattered field is assumed to be data for the problem. A time-harmonic dependence $e^{j\omega t}$ is assumed and suppressed.

The presence of targets in *D* is mathematically described by the contrast function $\chi \left(r\right)=\frac{\varepsilon \left(r\right)}{\varepsilon_{s}}-1$, which characterizes the target as an electromagnetic anomaly $\varepsilon \left(r\right)$ with respect to the soil’s properties.

The scattering phenomenon is described according the linear Born model [18]. Accordingly, the relationship between the measured scattered field $E_{s}$ and the unknown contrast function χ is written as

(1) $$E_{s}\left(r_{rx},r_{tx},\omega \right)=k_{0}^{2}\iint_{D}G\left(r_{rx},r,\omega \right)E_{i}\left(r,r_{tx},\omega \right)\chi \left(r\right)dr$$

where $k_{0}=\omega /c_{0}$ is the free-space wavenumber ($c_{0}$ = 3 × 10^8^ m/s is the speed of light in vacuum), $E_{i}$ is the incident field radiated from the source point $r_{tx}$ to r, and G is the external Green’s function describing the radiation from r to $r_{rx}$.

Due the proportionality between the electric field and the Green’s function, i.e.,

(2) $$E_{i}\left(r\prime ,r,\omega \right)=-j\omega \mu_{0}G\left(r,r\prime ,\omega \right)$$

where $\mu_{0}=4\pi \times 10^{-7}$ H/m and accounting for the Lorentz’ reciprocity principle [24], the integral Equation (1) can be reformulated as

(3) $$E_{s}\left(r_{rx},r_{tx},\omega \right)=\frac{jk_{0}}{Z_{0}}\iint_{D}E_{i}\left(r,r_{rx},\omega \right)E_{i}\left(r,r_{tx},\omega \right)\chi \left(r\right)dr$$

with $Z_{0}$ being the free-space impedance.

According to Equation (3), the kernel of the integral equation is completely characterized by the knowledge of the incident field radiated into the soil medium. Although an analytical (but not closed) expression for this field is available in the form of a spectral integral (see [18]), in this work we adopt a ray-based formulation. This approach offers clearer physical insight into the problem and serves as a foundation for the development of the equivalent permittivity model. It should be emphasized that the ray formulation is approximate, as it implicitly assumes far-field radiation; however, its accuracy has been demonstrated for observation distances above the air–soil interface as just comparable to the wavelength (see, e.g., [25]).

It must be also stressed that electromagnetic losses associated with the electrical conductivity of the soil, and possibly of the targets of interest, are only neglected in the model adopted to build the scattering operator to be inverted. This assumption is essential to describe the electromagnetic scattering in a simplified manner through a multi-frequency propagation model and does not regard the data (see Section 3.2). Moreover, neglecting the electrical conductivity in the imaging formulation does not impair an accurate localization and a rough estimate of geometrical features of the targets, as testified by the wide literature concerning field examples (see [21]).

### 2.1. Interface Reflection Point (IRP) Model

In this subsection, we briefly describe the interface reflection point (IRP) model recently proposed by the authors in [3]. As shown in Figure 1, a ray emitted by the source at $r_{tx}$ undergoes refraction at the IRP_t_ and impinges on the target. Then, the reflected ray is retransmitted in the air at IRP_r_ and reaches the observation point $r_{rx}$. According to [3] and neglecting unessential amplitude factors, the linear integral equation relating $E_{s}$ to χ is given by

(4) $$E_{s}\left(r_{rx},r_{tx},\omega \right)=-\frac{j\omega \sqrt{\varepsilon_{rs}}}{2\pi c_{0}}\iint_{D}\frac{T_{12}T_{21}}{\sqrt{\left(R_{t1}+R_{t2}\right)\left(R_{r1}+R_{r2}\right)}}e^{-jk_{0}\left(R_{t1}+R_{r1}+\sqrt{\varepsilon_{rs}}\left(R_{t2}+R_{r2}\right)\right)}\chi \left(r\right)dr=L_{IRP}[\chi ]$$

In the former equation, $T_{12}$ and $T_{21}$ are the Fresnel’s reflection coefficients at the air– soil and soil–air interface [24]; $R_{t1}$, $R_{t2}$, $R_{r1}$, and $R_{r2}$ are the lengths of the ray paths (see Figure 1); and $L_{IRP}\left(D\right)\to L^{2}(\Gamma \times \Omega )$ is a linear projection operator mapping the space of unknown onto the data space. Note that the Fresnel’s coefficients and the ray path lengths depend on the incidence angles $\left(\theta_{i}^{m},\theta_{i}^{n}\right)$ and the transmission angles $\left(\theta_{t}^{m},\theta_{t}^{n}\right).$

The IRP model defined by Equation (4) is considered hereafter as the reference model and used as benchmark to assess the performance of the EP model detailed in the following sections.

### 2.2. EP Model

The computational burden associated with the evaluation of $L_{IRP}$ is mainly due to the computation of IRP_t_ and IRP_r_. Indeed, for every combination of $r_{rx},r_{tx},$ and r, it is necessary to solve two non-linear equations to determine the angles $\theta_{i}^{m}$ and $\theta_{t}^{n}$ (see Figure 1), and consequently to evaluate the distances $R_{t1}$*,* $R_{r1}$*,* $R_{t2}$*,* and $R_{r2}$ appearing in the kernel of the integral Equation (4). These equations are omitted for brevity and their complete expressions can be found in [3].

To minimize the computational effort, an approximate ray-based formulation is adopted here based on the EP concept. Such a formulation describes the propagation in the two-layered medium as occurring within an effective medium characterized by a spatially varying dielectric permittivity. Note that the idea was originally proposed in [22] in relation to underwater imaging via GPR for a multi-monostatic configuration, and is applied here to the contactless MIMO GPR configuration (multiview/multistatic configuration).

As shown in Figure 1, the ray directed from the Tx antenna to the target and refracted at the IRP_t_ is replaced by a straight path of length $R_{t}=|{r-r}_{tx}|$ in an equivalent medium. In order to ensure equivalence between the two scenarios, it is necessary to match the overall phase variation along the ray path in the two-layered medium to the one in the equivalent medium, i.e.,

(5) $$k_{eqt}R_{t}=k_{0}(R_{t1}+\sqrt{\varepsilon_{rs}}R_{t2})$$

where $k_{eqt}=k_{0}\sqrt{\varepsilon_{eqt}}$ is the equivalent wavenumber on transmission and

(6) $$\varepsilon_{eqt}={\left(\frac{R_{t1}+\sqrt{\varepsilon_{rs}}R_{t2}}{R_{t}}\right)}^{2}$$

is the corresponding EP, which is obviously dependent on the location of the transmitting antenna.

A similar argument applied to the ray path from the target to the measurement point allows an EP at reception to be defined as

(7) $$\varepsilon_{eqr}={\left(\frac{R_{r1}+\sqrt{\varepsilon_{rs}}R_{r2}}{R_{r}}\right)}^{2}$$

where $R_{r}=|{r-r}_{rx}|$.

The EP formulas in (6) and (7) still need solving for the two refraction points as in the IRP model and, accordingly, they do not provide any advantage from the computation viewpoint. As in [22], we make the further assumption that the ray propagation occurs at normal incidence. Accordingly, two identical expressions for the EP are found:

(8) $$\varepsilon_{eqt}=\varepsilon_{eqr}\approx \varepsilon_{eq}={\left(\frac{h+\sqrt{\varepsilon_{rs}}z}{z+h}\right)}^{2}$$

It can be seen that $\varepsilon_{eq}$ no longer depends on the refraction point, but only on the depth *z*. In particular, $\varepsilon_{eq}$ is equal to one at the air–soil interface (*z* = 0) and approaches the soil’s relative permittivity $\varepsilon_{rs}\;$ for large values of *z*.

According to the EP model, the linear integral equation to be inverted can be written as

(9) $$E_{s}\left(r_{rx},r_{tx},\omega \right)=-\frac{j\omega \sqrt{\varepsilon_{rs}}}{2\pi c_{0}}\iint_{D}\frac{e^{-jk_{0}\sqrt{\varepsilon_{eq}}\left(R_{t}+R_{r}\right)}}{\sqrt{R_{t}+R_{r}}}\chi \left(r\right)dr=L_{EP}[\chi ]$$

where $L_{EP}$ is the linear operator defined according to the EP model.

It must be stressed that the kernel of the integral equation in (9) is now available in closed analytical form, which makes the evaluation of the operator much more efficient. However, the assumption of normally incident rays adopted for the computation of the phase terms inherently introduces a further approximation.

To quantify the error introduced by this approximation, we introduce the following residual phase error:

(10) $$\Delta \Phi \left(r_{tx},r_{rx},\omega ,r\right)=k_{0}\sqrt{\varepsilon_{eq}}\left(R_{t}+R_{r}\right)-k_{0}\left(R_{t1}+R_{r1}\right)-k_{0}\sqrt{\varepsilon_{rs}}\left(R_{t2}+R_{r2}\right)$$

The former equation expresses the phase difference along the Tx–target–Rx path between the EP model and the IRP model (reference). This quantity depends on $r_{tx}$, $r_{rx},$ ω, and r. Here, we consider a simpler and comprehensive representation of the error as a function of the generic point of the investigation domain, provided by the Mean Phase Error (MPE):

(11) $$MPE\left(r\right)=\frac{1}{MNP}\sum_{m=1}^{M}\sum_{n=1}^{N}\sum_{p=1}^{P}\left|\Delta \Phi \left(r_{tx,m},r_{rx,n},\omega_{p},r\right)\right|$$

where P denotes the number of samples in the frequency band Ω. The MPE in (11) provides a representation of the phase error over the considered investigation domain and the regions with the highest values are expected to provide worse reconstructions in terms of focusing and localization.

### 2.3. Data Inversion

The linear inverse problem defined by (3), and consequently by (4) and (9), is ill-posed [26]. Accordingly, a regularization strategy is necessary to obtain a physically meaningful solution in the presence of noise in data. In this work, we solve the inverse problem through the adjoint inversion [27]:

(12) $$\hat{\chi }=L^{+}E_{s}$$

with $L^{+}$ being the adjoint of *L.*

The spatial map $I=\left|\hat{\chi }\right|/max\left\{\left|\hat{\chi }\right|\right\}$, defined as the magnitude of the retrieved contrast function normalized to its maximum over *D*, denotes the tomographic image.

## 3. Numerical Analysis

### 3.1. PSF Analysis

**Figure 2:**
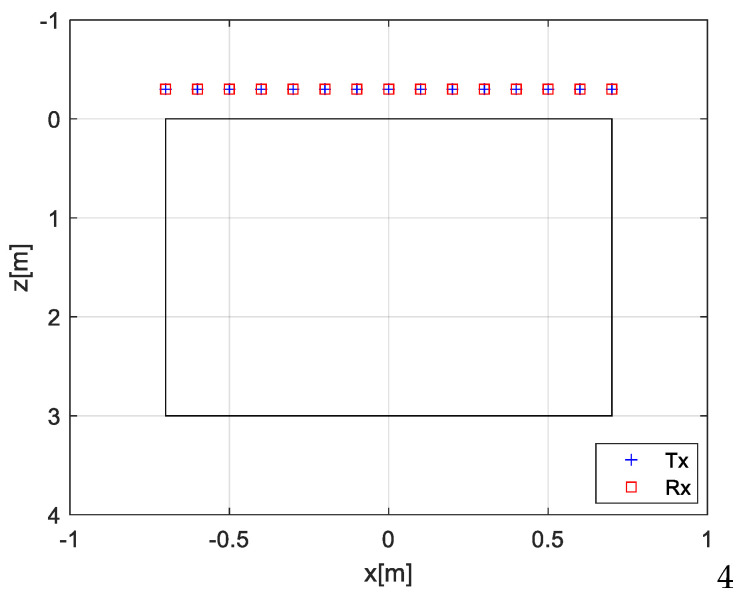
Simulated scenario. The investigation domain (black rectangle) is probed by *M* = *N =* 15 antennas.

This section presents numerical tests aimed at assessing the reconstruction performance of the EP-based inversion approach. The tests refer to the scenario depicted in Figure 2, where the investigation domain *D* = [−0.7, 0.7] × [0, 3] m^2^ is probed by M = 15 Tx and N = 15 Rx antennas uniformly spaced over the interval Γ = [−0.7, 0.7] m at a quota *h* = 0.3 m above the air–soil interface. The soil’s relative permittivity is $\varepsilon_{rs}=4$ and the antennas’ operating bandwidth [300, 900] MHz is sampled with a step of 10 MHz (61 frequencies). The investigation domain is discretized into square image pixels with sides of 0.025 m.

Scattered field data referring to a single point target placed at different locations in *D* are generated according to the IRP model in Equation (4) and inverted via (12) using both IRP and EP models. In this way, the PSF is obtained and the focusing performance achievable with both models is assessed.

**Figure 3:**
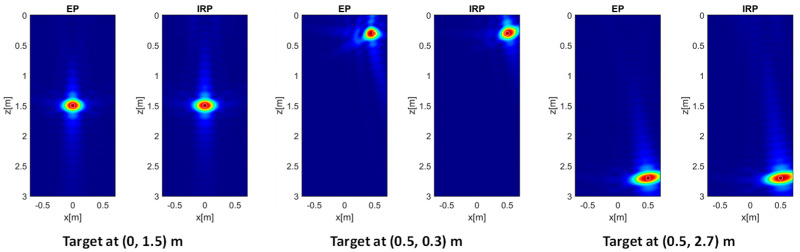
Normalized PSF amplitude for different point targets’ locations achieved with the EP and IRP models. $\varepsilon_{rs}=4$, *h* = 0.3 m, *M* = *N* = 15. The white circles denote the true targets’ positions. Color scale [0, 1].

Figure 3 shows the normalized amplitude of the PSF for a target located at (0, 1.5) m (left panels), (0.5, 0.3) m (middle panels), and (0.5, 2.7) m (right panels). Specifically, the EP model produces reconstructions almost identical to those obtained with the IRP model. However, for the shallower and laterally positioned target at (0.5, 0.3) m, the reconstruction provided by EP model inversion appears slightly defocused and exhibits minor target delocalization.

**Figure 4:**
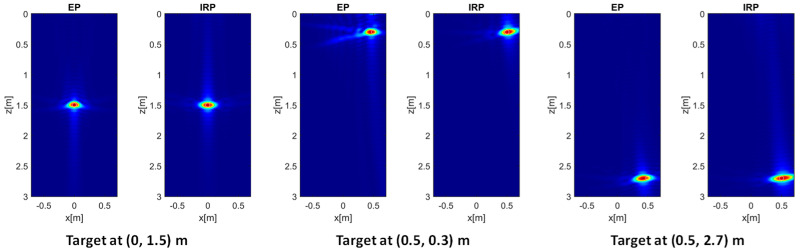
Normalized PSF amplitude for different point targets’ locations achieved with the EP and IRP models. $\varepsilon_{rs}=13$, *h* = 0.3 m, *M* = *N* = 15. The white circles denote the true targets’ positions. Color scale [0, 1].

The case of higher soil permittivity ($\varepsilon_{rs}=13$) and representative of moist soil conditions is analyzed next. It is interesting to observe from the images in Figure 4 that the EP model still provides satisfactory results close to those obtained with the reference IRP model, particularly when the target is centrally located (left panels). However, when the target is shallow and laterally displaced (middle panels), the PSF obtained with the EP model is characterized by stronger sidelobes, as revealed by more pronounced tails surrounding the main lobe. In the case of laterally displaced and deeper targets (right panels), the EP model yields acceptable results that are consistent with those of the IRP model.

It is worth noting that the PSFs in Figure 4, compared to the corresponding ones in Figure 3, exhibit an improved resolution along the *z* direction. This behavior is expected, since it can be shown that the depth resolution is inversely proportional to $\sqrt{\varepsilon_{rs}}$ following an approximate derivation similar to that reported in [18] for the case of a monostatic GPR configuration.

**Figure 5:**
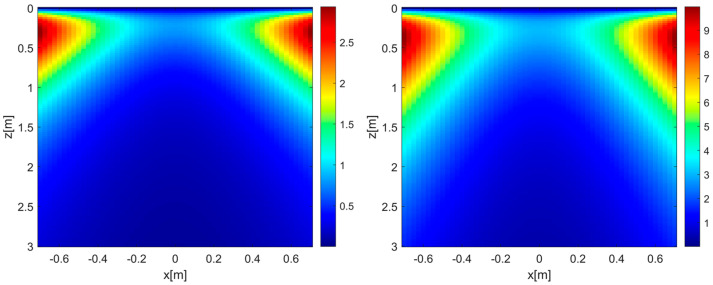
MPE achieved for $\varepsilon_{rs}$ = 4 (**left panel**) and $\varepsilon_{rs}$ = 13 (**right panel**). *h* = 0.3 m, *M* = *N* = 15. The color scales are expressed in radians.

To better interpret the above reconstruction results based on the EP model, it is useful to examine the phase error evaluated according to (11). To this end, the left and right panels in Figure 5 show the MPE over the investigation domain for $\varepsilon_{rs}$ = 4 (left panel) and $\varepsilon_{rs}$ = 13 (right panel), respectively, with all remaining parameters kept constant. It is interesting to observe that, when $\varepsilon_{rs}=4$, the phase error is very low in most of the investigation domain and only increases in its shallower and lateral regions. This explains the minor defocusing previously observed for the target at (0.5, 0.3) m (see middle panel of Figure 3). On the other hand, an increase in the phase error arises in those regions when $\varepsilon_{rs}=13$, which justifies the sidelobe level growth seen in the middle panel of Figure 4.

**Figure 6:**
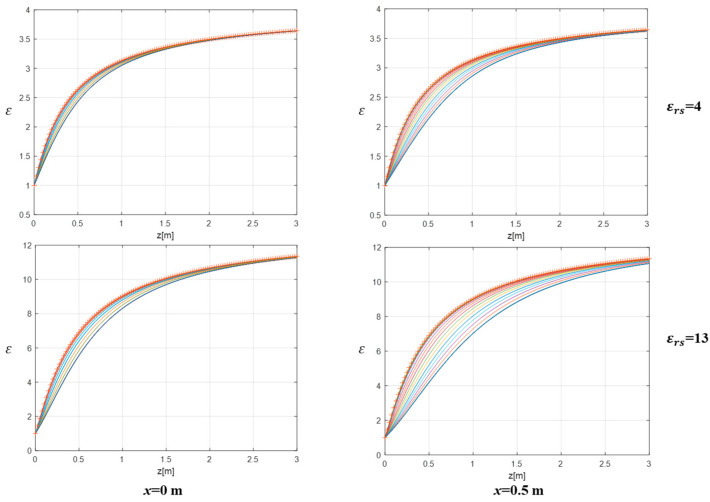
Trends of equivalent permittivity $\varepsilon_{eqt}$ and $\varepsilon_{eqr}$ (colored curves), $\varepsilon_{eq}$ (red symbols) versus depth at *x* = 0 (**left panels**) and *x* = 0.5 m (**right panels**) for $\varepsilon_{rs}=4$ (**top panels**) and $\varepsilon_{rs}=13$ (**bottom panels**). *h* = 0.3 m, *M* = *N* = 15.

The accuracy of the EP model approximation can be evaluated from a different perspective by comparing the exact values of the equivalent permittivities $\varepsilon_{eqt}$ (in transmission) and $\varepsilon_{eqr}$ (at reception), defined in (6) and (7), with the approximated equivalent permittivity $\varepsilon_{eq}$ in (8) involved in the EP inversion model. In this respect, Figure 6 shows the trends of $\varepsilon_{eqt}$ and $\varepsilon_{eqr}$ (colored curves) and $\varepsilon_{eq}$ (red symbols) versus depth at *x* = 0 m (left panels) and *x* = 0.5 m (right panels) for $\varepsilon_{rs}=4$ (top panels) and $\varepsilon_{rs}=13$ (bottom panels). As can be observed, all curves are unitary at the air–soil interface and tend toward the soil’s permittivity $\varepsilon_{rs}$ for large *z*. However, the $\varepsilon_{eq}$ curves provide a better approximation of $\varepsilon_{eqt}$ and $\varepsilon_{eqr}$ at the center of the investigation domain (*x* = 0) and for the lower permittivity value $\varepsilon_{rs}=4$ (top left panel). Conversely, in agreement with previous observations, the worst approximation occurs laterally (i.e., at *x* = 0.5 m) when$\;\varepsilon_{rs}=13$ (bottom-right panel) because the curves exhibits a large deviation.

A quantitative assessment of the focusing performance is provided by the entropy metric defined as [28]

(13) $$E=-\sum_{q=1}^{Q}\frac{I_{q}^{2}}{\sum_{q=1}^{Q}I_{q}^{2}}\ln\left[\frac{I_{q}^{2}}{\sum_{q=1}^{Q}I_{q}^{2}}\right]$$

where Q is the total number of pixels in the tomographic image *I*. The entropy *E* in (13) quantifies the focusing quality of the point target, taking larger values for an increased blurring of the tomographic image. Note that *E* becomes zero in the ideal case of a point target reconstructed by a single bright pixel.

The entropy values referring to the considered target positions and permittivity values for both EP and IRP models are summarized in Table 1. These data agree with previous findings as they confirm that the entropy with the EP model is higher than the IRP model’s entropy when the target is located at (0.5, 0.3) m, and the largest entropy increase (i.e., from 4.5 to 5.0) is attained when $\varepsilon_{rs}=13$. It is worth noting that the entropy *E* attains lower values for increasing values of $\varepsilon_{rs}$ due to the better resolution achievable along z.

**Figure 7:**
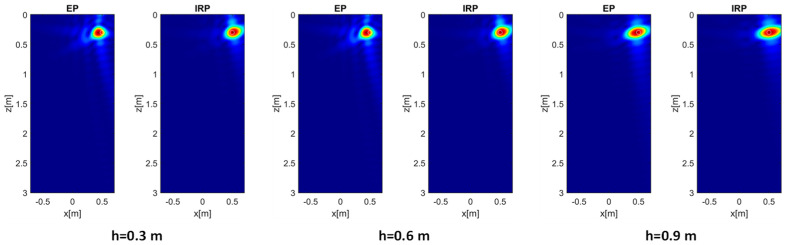
Normalized PSF amplitude for the target at (0.5, 0.3) m and different *h* values achieved with the EP and IRP models. $\varepsilon_{rs}=$ 4, *M* = *N* = 15. The white circles denote the true targets’ positions. Color scale [0, 1].

In the following analysis, the soil’s permittivity $\varepsilon_{rs}$ is fixed at a value of 4 and the target’s position is set to (0.5, 0.3) m, which represents the most challenging scenario of a shallow and laterally displaced target. The goal of the analysis is to investigate the effect of increasing the measurement height *h* on the reconstruction capability of the EP model. Therefore, in addition to *h* = 0.3 m, the cases *h* = 0.6 and *h* = 0.9 m are also considered. The reconstructions displayed in Figure 7 correspond to the aforementioned cases and confirm that the EP model provides results that are in good agreement with those of the IRP model, except for a slight increase in the sidelobe level. However, the achieved entropy values (here omitted for brevity) obtained in the different cases are essentially equivalent.

**Figure 8:**
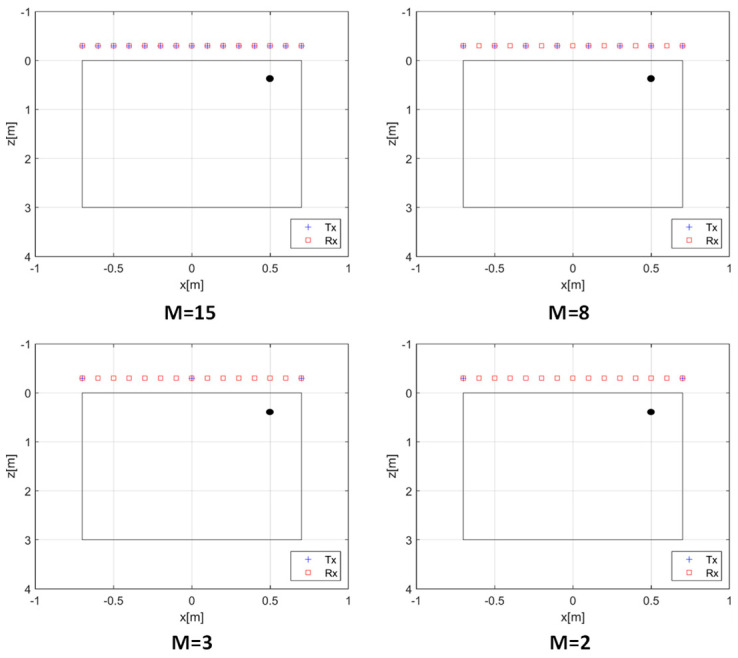
Scenarios with a progressively decreasing number of Tx antennas. The black circle denotes the point target at (0.5, 0.3) m.

The analysis of the EP model imaging performance is also performed by investigating the effect of the number of transmitters *M*, while keeping all other parameters fixed. In particular, the soil’s permittivity is set to 4, the measurement height is *h* = 0.3 m, the target is located at (0.5, 0.3) m, and *N* = 15 measurement points are considered. The Tx antennas are assumed to be equally spaced over Γ and progressively reduced in number (*M* = 15, 8, 3, 2), as represented in the scenarios of Figure 8.

**Figure 9:**
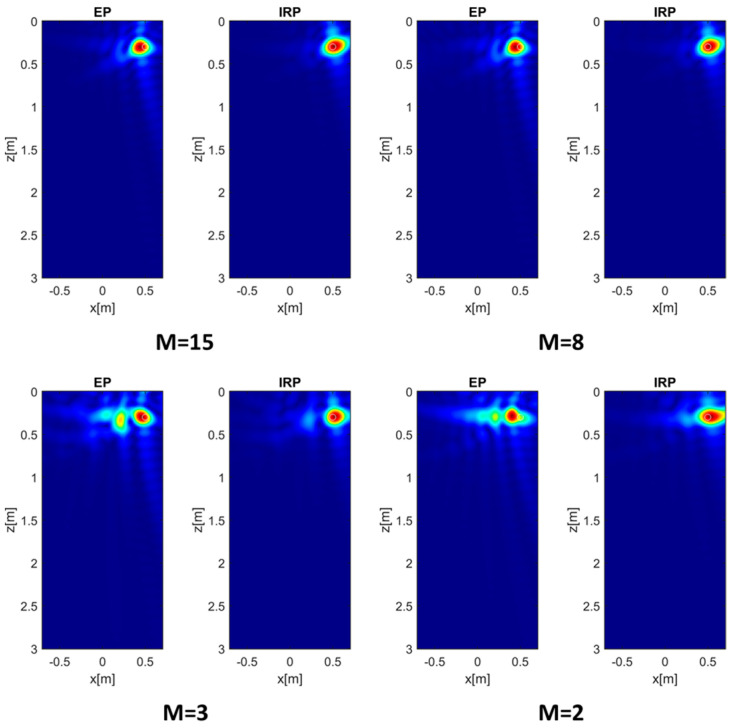
Normalized PSF amplitude for the target at (0.5, 0.3) m and different *M* values achieved with the EP and IRP models. $\varepsilon_{rs}=$ 4, *h* = 0.3 m, and *N* = 15. The white circles denote the true targets’ positions. Color scale [0, 1].

The reconstructions obtained for different values of *M* are reported in Figure 9. It is interesting to note that, for *M* = 15 and 8, the EP model provides reconstructions that are only slightly less focused than those obtained with the IRP model. However, when the number of transmitters is significantly reduced (*M* = 3 and 2), the image obtained using the EP model is characterized by the presence of a spurious target close to the true target, thus degrading the overall reconstruction quality in terms of the signal-to-clutter ratio.

**Figure 10:**
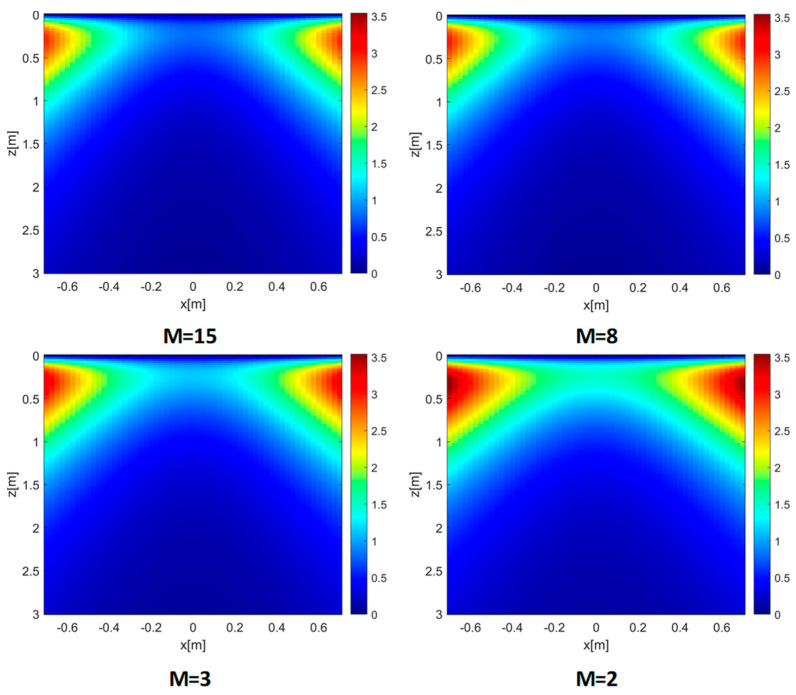
MPE versus *M.* $\varepsilon_{rs}=4$, *h* = 0.3 m, *N* = 15. The color scales are expressed in radians.

The former observations are confirmed by the MPE in Figure 10, which shows a general increase in the phase error as *M* is reduced. In addition, the entropy values reported in Table 2 grow (i.e., worse in terms of focus) when *M* decreases and reveal a higher degradation of the EP model performance for low values of *M*.

In the following analysis, we study the effect of the number of Rx antennas *N* when the number of Tx antennas is fixed at *M* = 15, $\varepsilon_{rs}$ = 4, and *h* = 0.3 m. The entropy values listed in Table 3 suggest that the EP model performs worse than the IRP model, especially when the number of Rx antennas is progressively reduced. Conversely, the IRP models turns out to be insensitive to the variations in the number of receivers.

### 3.2. Extended Target Reconstructions

This subsection reports the results of some reconstructions aiming to test the effectiveness of the EP inversion approach in the case of extended targets. To this end, full-wave data are generated by using the electromagnetic solver GPRMAX [29] based on the Finite Difference Time Domain (FDTD) technique [30]. A scenario with the antennas located at a measurement height of *h* = 0.3 m is considered. The antennas radiate a Ricker pulse with a center frequency of 600 MHz, and the duration of the observation time window is 60 ns. The soil is modeled as a medium with relative permittivity $\varepsilon_{rs}=4$ and electrical conductivity $\sigma_{s}=$ 1 × 10^−4^ S/m. The investigation domain *D* = [−0.7, 0.7] × [0, 3] m^2^ located in the soil contains two targets: (i) a circular cavity with a radius of 0.25 m and centered at (0, 1.5) m; and (ii) a granite rock ($\varepsilon_{rt}$ = 5.45, $\sigma_{t}$ = 7 × 10^−8^ S/m) [31] with dimensions 0.3 × 0.1 m^2^ and centered at (0.5, 0.35) m.

The time-domain total field data (raw radargrams) undergo preprocessing before the focusing step [3]. In detail, the two-way travel time corresponding to the zero depth in the tomographic image is first set as $t_{0}$ = 1.7 ns. After this, a space-varying time-gating operation is applied, setting the early time response in the radargram to zero, thereby removing the direct (air) wave and the first signal reflection from the air–soil interface. To this end, the following formula is applied to determine the gating time:

(14) $$t_{gat}=t_{0}+\frac{\sqrt{{\left(2h\right)}^{2}+{\left(x_{tx}-x_{rx}\right)}^{2}\;}}{c_{0}}+\delta$$

where δ= 1 ns is a time delay accounting for the duration of the Ricker pulse.

The achieved scattered field radargrams are transformed into the frequency domain over the band [300, 900] MHz with a step of 10 MHz, and undergo adjoint-based inversion.

**Figure 11:**
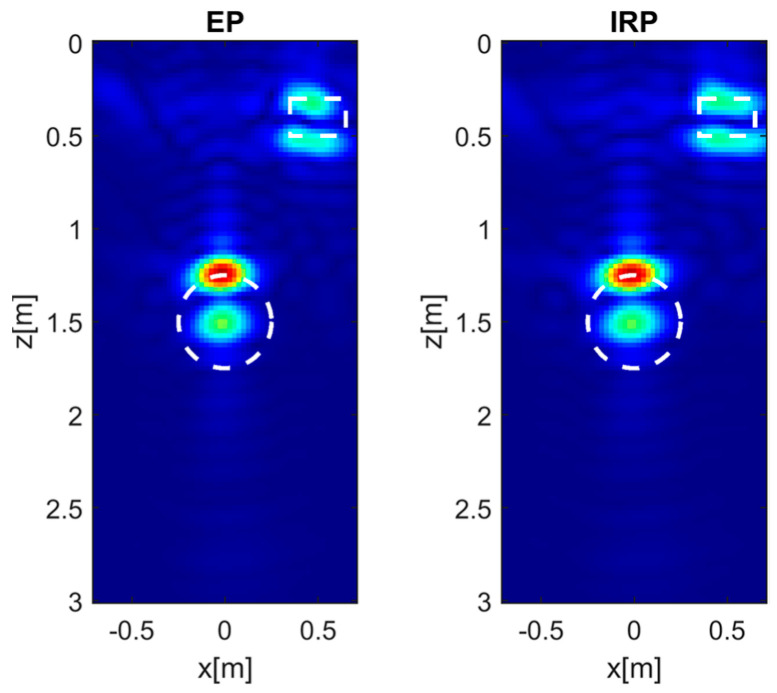
Tomographic reconstructions of two extended targets achieved with EP and IRP models. The data are simulated by assuming $\varepsilon_{rs}$ = 4, $\sigma_{s}$ = 1 × 10^−4^ S/m, *h* = 0.3 m, and *M* = *N* = 15. The white dashed lines denote the true targets. Color scale [0, 1].

The images in Figure 11 show the reconstructions of the two targets obtained using the EP and IRP models for $\varepsilon_{rs}=4$, *h* = 0.3 m and *M* = *N* = 15. As can be observed, the reconstructions are very similar, particularly with regard to the cavity, whereas the reconstruction of the rock exhibits only minor differences between the two models since the IRP model seems to provide a superior reproduction of the target contour. It is worth noting that, due to the reflection-mode measurement configuration, the retrievable spectral support of the unknown exhibits a bandpass behavior along the depth direction [18]. Accordingly, only the upper and lower target edges can be retrieved. In addition, the LMWT approach assumes a wave propagation velocity inside the targets equal to that of the surrounding soil. As a result, the bottom edges of both targets are delocalized; this effect has been deeply investigated in the literature [32]. In this respect, the most pronounced delocalization occurs for the circular cavity, which is characterized by a higher dielectric contrast (and lower wave velocity) with respect to the soil medium.

**Figure 12:**
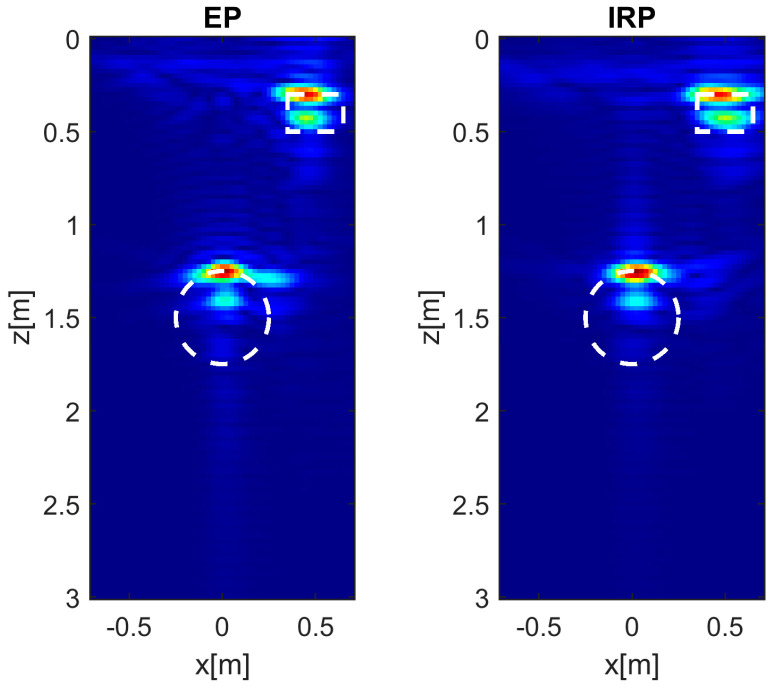
Tomographic reconstructions of two extended targets achieved with EP and IRP models. The data are simulated by assuming $\varepsilon_{rs}$ = 13, $\sigma_{s}$ = 1 × 10^−4^ S/m, *h* = 0.3 m, and *M* = *N* = 15. The white dashed lines denote the true targets. Color scale [0, 1].

The reconstructions plotted in Figure 12 are achieved when $\varepsilon_{rs}=$ 13, *h* = 0.3 m, and M=N=15. In this case, as observed in the PSF analysis reported in Section 3.1, the higher soil permittivity makes the target reconstructions obtained with the EP model slightly cluttered and the object contours are less accurately reproduced. Nevertheless, the reconstructions remain very satisfactory from a practical viewpoint.

**Figure 13:**
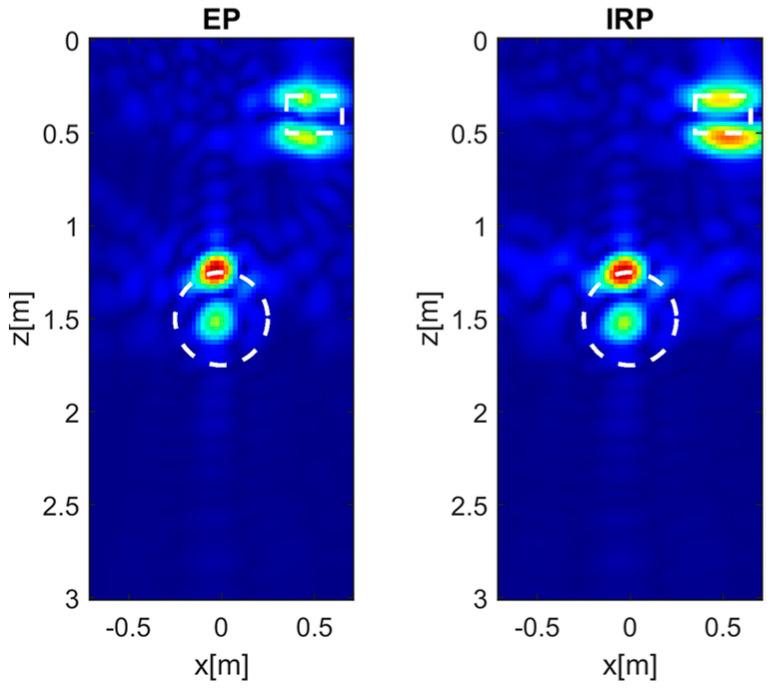
Tomographic reconstructions of two extended targets achieved with EP and IRP models. The data are simulated by assuming $\varepsilon_{rs}=$ 4, $\sigma_{s}=$ 1 × 10^−4^ S/m, *h* = 0.3 m, and *M* = 2, *N* = 15. The white dashed lines denote the true targets. Color scale [0, 1].

**Figure 14:**
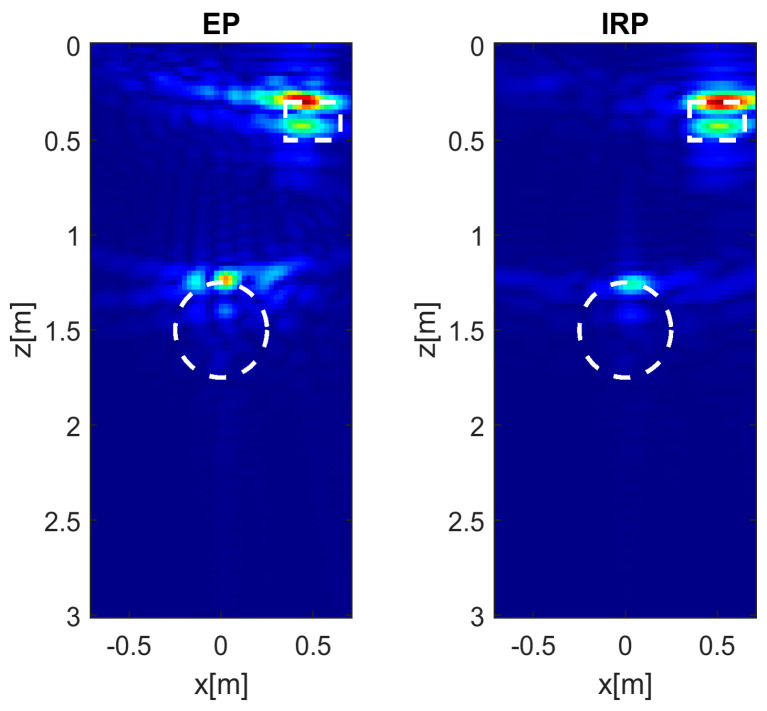
Tomographic reconstructions of two extended targets achieved with EP and IRP models. The data are simulated by assuming $\varepsilon_{rs}$ = 13, $\sigma_{s}$ = 1 × 10^−4^ S/m, *h* = 0.3 m, and *M* = 2, *N* = 15. The white dashed lines denote the true targets. Color scale [0, 1].

Figure 13 and Figure 14 display the reconstructions with a reduced number of Tx antennas (*M* = 2) when $\varepsilon_{rs}$ = 4 and 13, respectively. Even for $\varepsilon_{rs}$ = 4, the results provided by the EP model are satisfactory. On the other hand, when $\varepsilon_{rs}$ = 13, a non-negligible increase in the clutter is observed, consistent with previous observations in Figure 9 for *M* = 2.

Note that the shallower object has a permittivity ($\varepsilon_{rt}$ = 5.45) close to that of the soil when $\varepsilon_{rs}$ = 4; as a result, its lower boundary is quite well localized (Figure 11 and Figure 13). This does not occur when $\varepsilon_{rs}$ = 13 (Figure 12 and Figure 14).

**Figure 15:**
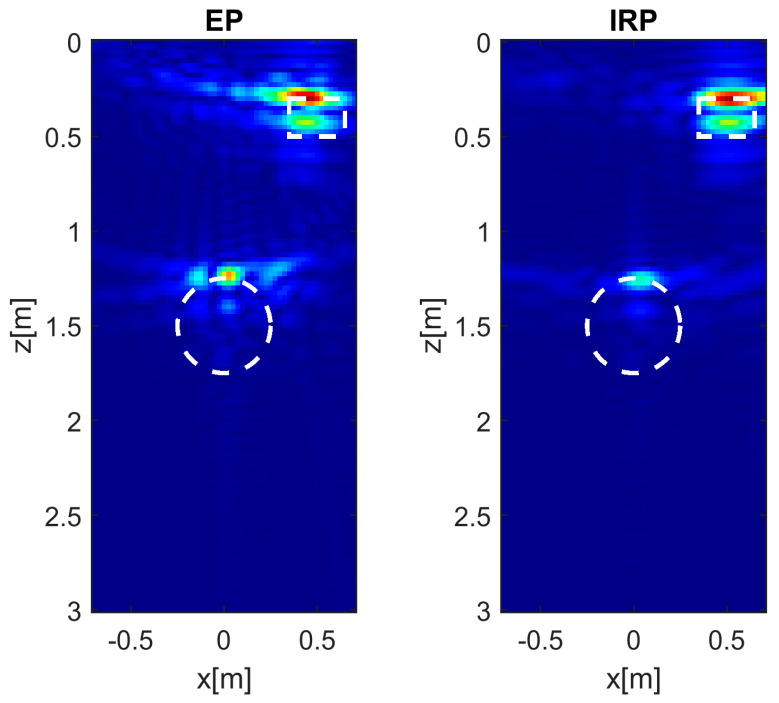
Tomographic reconstructions of two extended targets achieved with EP and IRP models. The data are simulated by assuming $\varepsilon_{rs}$ = 13, $\sigma_{s}$ = 1 × 10^−2^ S/m, *h* = 0.3 m, and *M* = 2, *N* = 15. The white dashed lines denote the true targets. Color scale [0, 0.6].

The numerical test associated with Figure 14 is repeated by generating data for higher soil conductivity ($\sigma_{s}$ = 1 × 10^−2^ S/m) in order to highlight the effect of the increased signal attenuation on the reconstruction quality. The results in Figure 15 confirm that both targets can still be reconstructed using the EP model, although with reduced quality compared to the IRP image. Note that both images are now displayed using the color scale [0, 0.6] to emphasize the response from the deeper target, which is weakened due to the increased soil attenuation and the lack of gain applied to the data.

Finally, Table 4 compares the computation times required by the EP and IRP reconstruction approaches. In this respect, it must be stressed that the computational load is mostly linked to the construction of the scattering operator, which requires resolving the reflection points at the air–soil interface within the IRP approach. On other hand, the adjoint inversion based on (12) has a negligible impact since it involves a simple matrix–vector multiplication.

The imaging algorithms were implemented under the MATLAB 2023a environment on a desktop PC equipped with an INTEL XEON GOLD 6136 CPU (3.0 GHz clock frequency) and 256 GB RAM. The computation times reported in Table 4 as a function of *M* demonstrate that the EP inversion approach offers a substantial reduction in the computational load with respect to the IRP approach, with a more significant gain as the number of transmitters increases.

## 4. Conclusions

This work presented a 2D linear microwave tomography approach for contactless MIMO-GPR based on the equivalent permittivity concept. By incorporating a depth-dependent equivalent permittivity in a ray-based propagation model, the method accounts for the electromagnetic wave propagation through the air–soil interface, enabling a computationally efficient linear inversion, also thanks to the adjoint scattering operator. The applicability of the approach was investigated by comparing the imaging results with those provided by the IRP-model-based approach previously proposed by the authors. The imaging results referring to both point-like and extended targets revealed that the EP-based approach provides sufficiently accurate target reconstructions compared to the reference IRP model. This claim is corroborated by the phase error analysis and by image entropy, which was used as a metric to assess the reconstruction accuracy. However, a slight reduction in focusing performance was observed for high soil permittivity and a few transmitting antennas due to the hypothesis of normal incidence. The presented results confirm that this imaging approach is suitable for practical applications given the satisfactory results and substantial computational savings.

It is worth pointing out that an extension of the proposed approach to the 3D case is feasible provided that MIMO data are collected over a planar surface, for instance, by moving the antenna array. Furthermore, the formulation of the 3D imaging is not expected to be particularly complex from a signal processing perspective if the depolarization effects are neglected in the scattering model adopted for the imaging.

Future research activities will compare the presented approach with other state-of-the-art qualitative imaging techniques.

## Figures and Tables

**Table 1:** Entropy values for different target locations and soil permittivity values. *h* = 0.3 m, *M* = 15, and *N* = 15.

Target Position	$\varepsilon_{rs}$	EP	IRP
(0.5, 0.3) m	4	5.2	5.0
(0, 1.5) m	4	5.2	5.2
(0.5, 2.7) m	4	5.5	5.5
(0.5, 0.3) m	13	5.0	4.5
(0, 1.5) m	13	4.5	4.5
(0.5, 2.7) m	13	4.8	4.8

**Table 2:** Entropy values versus *M* achieved with the EP and IRP inversion approaches. $\varepsilon_{rs}=$ 4, *h* = 0.3 m, *N* = 15.

*M*	EP	IRP
15	5.2	5.0
8	5.2	5.0
3	6.0	5.3
2	6.1	5.4

**Table 3:** Entropy values versus *N* achieved with the EP and IRP inversion approaches. $\varepsilon_{rs}=$ 4, *h* = 0.3 m, *M* = 15.

*N*	EP	IRP
15	5.2	5.0
8	5.2	5.0
3	5.8	5.0
2	5.8	5.0

**Table 4:** Computation times [s] for EP and IRP inversion approach vs. *M* for *N* = 15.

*M*	EP	IRP
15	2.79	17.51
8	1.52	12.48
3	0.55	8.85
2	0.40	8.18

## Data Availability

The raw data supporting the conclusions of this article will be made available by the authors on request.
